# Electrocatalyst/Semiconductor Interfaces in Photoelectrocatalysts: Mechanistic Analysis of the Impact of Nanoparticle Electrocatalyst Coverage on Performance

**DOI:** 10.1002/adma.74478

**Published:** 2026-08-04

**Authors:** Aarti Mathur, Ahmet Sert, Suljo Linic

**Affiliations:** ^1^ Department of Chemical Engineering University of Michigan Ann Arbor Michigan USA; ^2^ Catalysis Science and Technology Institute University of Michigan Ann Arbor Michigan USA

**Keywords:** photocatalysis, nanoscience, semiconductors

## Abstract

Interfaces between metal nanoparticle electrocatalysts (np‐EC) and semiconductors (SC) play a key role in photovoltage generation in photoelectrochemical np‐EC/SC water splitting systems. In this contribution, we investigate how the coverage of np‐EC on SC influences the performance of photoelectrodes. We focus on a case study of np‐Ni/n‐Si photoelectrocatalysts for the oxygen evolution reaction (OER). By systematically varying Ni coverage, we show that np‐EC coverage not only impacts light absorption and electrocatalytic activity but also governs the chemical evolution of the EC/SC interface under operating conditions by dictating the terminal thickness of interfacial insulating SiO_x_ layers that form during system operation. We demonstrate that this evolution of the interfacial SiO_x_ ultimately governs the system performance. We shed light on the underlying processes that govern the rate of growth for these interfacial oxide layers and describe how catalyst coverage impacts these processes. These findings highlight reaction environment‐induced changes to the EC/SC interface as a critical parameter that affects the performance of photoelectrochemical systems.

## Graphical abstract

Reducing Ni nanoparticle coverage on n‐Si photoanodes enhances photovoltage by promoting thicker interfacial SiO_x_ formation during photoelectrochemical oxygen evolution reaction (OER). Experiments, analytical modeling, and finite‐element simulations reveal that coverage‐dependent chemical interface evolution, rather than geometric effects alone, governs charge recombination and photoelectrochemical water oxidation performance, informing interface design strategies.

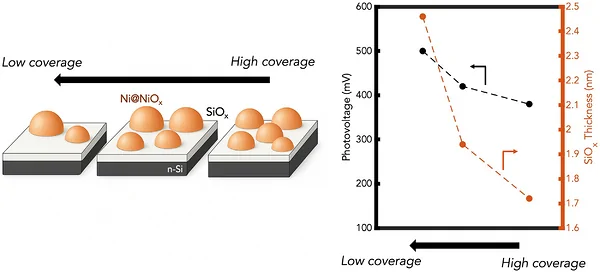

## 1 Introduction

Photoelectrochemical water splitting, employing metal nanoparticle electrocatalysts (np‐EC) coupled with semiconductor (SC) photoelectrodes, is a promising approach to produce renewable hydrogen [1]. The performance (i.e., photovoltage) of such photoelectrodes is largely governed by the chemical and physical nature of the nanoparticle electrocatalyst/semiconductor (np‐EC/SC) interfaces [2, 3]. In contrast to buried junction architectures in which a continuous and impermeable EC film protects the EC/SC interface, in photoelectrochemical systems that employ np‐EC/SC junctions these interfaces are typically unburied and therefore directly exposed to the reactive electrolyte. As a result, reaction‐induced changes to the EC/SC interface are particularly pronounced in np‐EC/SC systems. For example, it has been established that for some SCs with a desirable band gap, these np‐EC/SC interfaces can oxidize and degrade under reaction conditions (e.g., during oxygen evolution reaction (OER)) [4, 5, 6, 7]. On the other hand, for other SCs (e.g., Si), surface oxidation leads to the passivation of the SC, protecting the underlying SC from further corrosion and therefore improving the system stability [8, 9].

In addition to affecting stability, these reaction‐induced changes to the np‐EC/SC interface can affect the performance of these systems [10]. For example, we recently showed that spontaneously evolved interfacial SiO_x_ layers lead to significantly enhanced photovoltages for photoanodes deploying Ni nanoparticles anchored on n‐Si (np‐Ni/n‐Si) relative to their buried junction counterparts that deploy planar films of Ni covering n‐Si [11, 12]. We showed that the reaction environment‐induced formation of the insulating SiO_x_ layer in the unburied junction system in the aqueous electrolyte reduces e^−^‐h^+^ recombination rates by limiting the rates of the majority charge carriers reaching the Ni EC under illumination [13, 14, 15, 16, 17, 18].

In this study, we examine the impact of nanoparticle EC coverage on the evolution of these EC/SC interfaces and on the overall performance of these photoelectrodes. Our objective is to determine how nanoparticle EC coverage on SC affects key performance metrics (i.e., photocurrent and photovoltage), as well as to uncover the underlying physical and chemical processes governing these interactions. We address these questions by focusing on the model study of electrodeposited np‐Ni EC on n‐Si light absorber in photoelectrochemical OER. We find that systems with varied np‐Ni EC coverage on n‐Si exhibit different performance (i.e., generate a different photovoltage). We analyze the mechanisms governing the operation of these systems and show that the terminal thickness of the insulating interfacial SiO_x_—formed at the EC/SC interface in aqueous electrolyte—strongly depends on the np‐EC coverage. Specifically, we show that systems with lower np‐EC coverage tend to develop thicker SiO_x_ layers. This terminal oxide thickness largely dictates the photovoltage generated by these systems. We also shed light on the reasons behind the different terminal thickness of these interfacial SiO_x_ layers as a function of the np‐EC coverage. Together, these results indicate that chemical changes to the interface under reaction conditions must be carefully considered when designing photoelectrochemical systems and devices.

We note that previous studies have ascribed the differences in the performance of np‐Ni/n‐Si photoelectrodes to strictly physical factors, such as the pinch‐off effect [19, 20, 21], without invoking chemical changes to the interface [9, 22, 23, 24]. These prior works have attributed photovoltage differences across varying EC particle size and coverage to geometric or electrostatic effects local to the EC/SC interface, whereby regions of high barrier height near the nanoparticle perimeter dominate the device photovoltage. While our results are consistent with the observation that EC coverage influences photovoltage, we find that this relationship is more directly governed by the thickness and quality of the interfacial SiO_x_ layer, which varies systematically with Ni/NiO_x_ nanoparticle coverage under electrochemical operating conditions.

## 2 Characterization of Photoanodes With Varied Nanoparticle Coverage Regimes

We fabricated and analyzed three experimental systems with electrodeposited Ni nanoparticles on BHF‐etched n‐Si (np‐Ni/n‐Si) (see Experimental Section and Figure S1 for details). Electrocatalyst coverage was quantified based on top‐down scanning electron microscope (SEM) images of each system. The properties of np‐Ni on Si for the three systems were: (1) ∼20 nm (± 1.7 nm) average particle radius with ∼9% (± 2.1%) catalyst coverage, (2) ∼20 nm (± 8.5 nm) average particle radius with ∼15% (± 1.2%) catalyst coverage, and (3) ∼30 nm (± 0.4 nm) average particle radius with ∼25% (± 1.2%). Figure 1b–d shows SEM images of representative low, medium, and high coverage samples, respectively. See Table S1 for a complete list of parameters and standard deviations.

**FIGURE 1:**
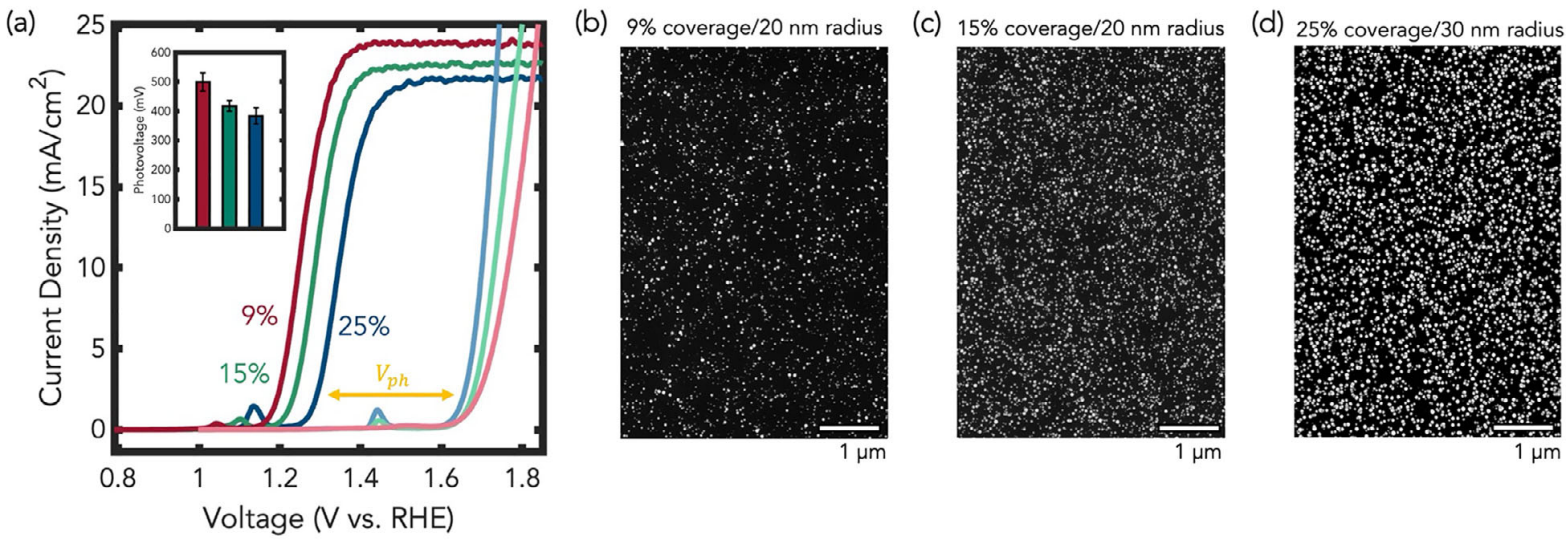
(a) Photoelectrochemical (solid lines) and dark electrochemical (faint lines) forward‐going OER LSV data for np‐Ni/n‐Si samples with varied coverage in 1 M KOH. Inset shows the measured photovoltage (at 1 mA/cm^2^) for each coverage. All data shown after 30+ cycles of activation under 1‐sun illumination intensity. The percentage markers on each bar indicate the top–down coverage of the sample, and the coloring corresponds to the data in (a). Inset error bars are obtained from the variation of a minimum of three independent measurements. (b‐d) SEM micrographs of (b) 9%, (c) 15%, and (d) 25% coverage samples. See Figures S1–S4 for extended characterization data.

The data in Figure 1a show representative linear sweep voltammograms (LSV) of each system under 1‐sun AM 1.5G illumination measured for photoelectrocatalytic water oxidation (i.e., OER). The data also show the LSV curves for np‐Ni/p^+^‐Si control samples, containing approximately identical coverage and size of np‐Ni on p^+^‐Si (see Supporting Information for details), measured in dark electrocatalytic experiments. The experimental photovoltage (inset in Figure 1a) was determined by subtracting the onset potentials (i.e., the voltage of each sample at 1 mA cm^−2^) for p^+^‐Si control samples, tested in the dark, from the counterpart n‐Si samples tested under 1‐sun illumination.

The data in Figure 1a show that the dark electrocatalytic activity decreases at lower coverages, with higher overpotentials required to reach the OER onset potential as the np‐Ni coverage is decreased from 25% to 9%. The observed changes in the dark onset potential are expected since lower coverages result in a lower number of catalytically active sites available to execute OER [25]. Surprisingly, the data in Figure 1a also show that for the illuminated n‐Si samples, the OER onset potential improves for lower np‐Ni coverages. In addition, the data also show that the photo‐limiting current increases for lower coverages, which is also expected as a lower loading of np‐Ni leads to less parasitic light absorption and reflection by Ni [26].

The data in Figure 1a inset show the photovoltages measured for each system using the LSV data from Figure 1a. The sample with 9% np‐Ni coverage generated the largest photovoltage, which was measured to be 498 ± 31 mV. The sample with 15% coverage generated 417 ± 18 mV of photovoltage, while the 25% sample generated 383 ± 28 mV. These data show that by reducing the catalyst coverage from 25% to 15%, ∼ 40 mV of additional photovoltage was generated, and by reducing the catalyst coverage further down to 9%, an additional ∼ 80 mV of photovoltage was gained. Overall, going from 25% to 9% coverage, we measured a total photovoltage improvement of ∼ 120 mV.

To characterize the systems further, we performed cross‐sectional STEM imaging and EDS elemental mapping on the used photoelectrodes. STEM‐EDS mapping in Figure 2a–c shows that each system had a similar interfacial and surface chemical profile, with an evolved NiO_x_ shell around the Ni nanoparticle (of similar thickness across samples) and an interfacial SiO_x_ layer [20]. To determine the precise thickness of interfacial SiO_x_ for each system, we performed atomic‐resolution STEM characterization on each sample after potential cycling (i.e., 30+ cycles). These STEM images are shown in Figure 2d–f for representative low‐, medium‐, and high‐coverage samples, respectively. Images taken at multiple points across a sample were used to determine the average interfacial SiO_x_ thickness for each system studied herein. This analysis revealed that the 9% coverage sample had 2.46 ± 0.16 nm of interfacial SiO_x_, while the 15% sample had 1.94 ± 0.18 nm and 25% had 1.72 ± 0.13 nm, respectively. These results show that even though these samples were fabricated under identical conditions, after 30+ cycles of photoelectrochemical testing, they surprisingly exhibit significant differences in the terminal interfacial SiO_x_ thickness.

**FIGURE 2:**
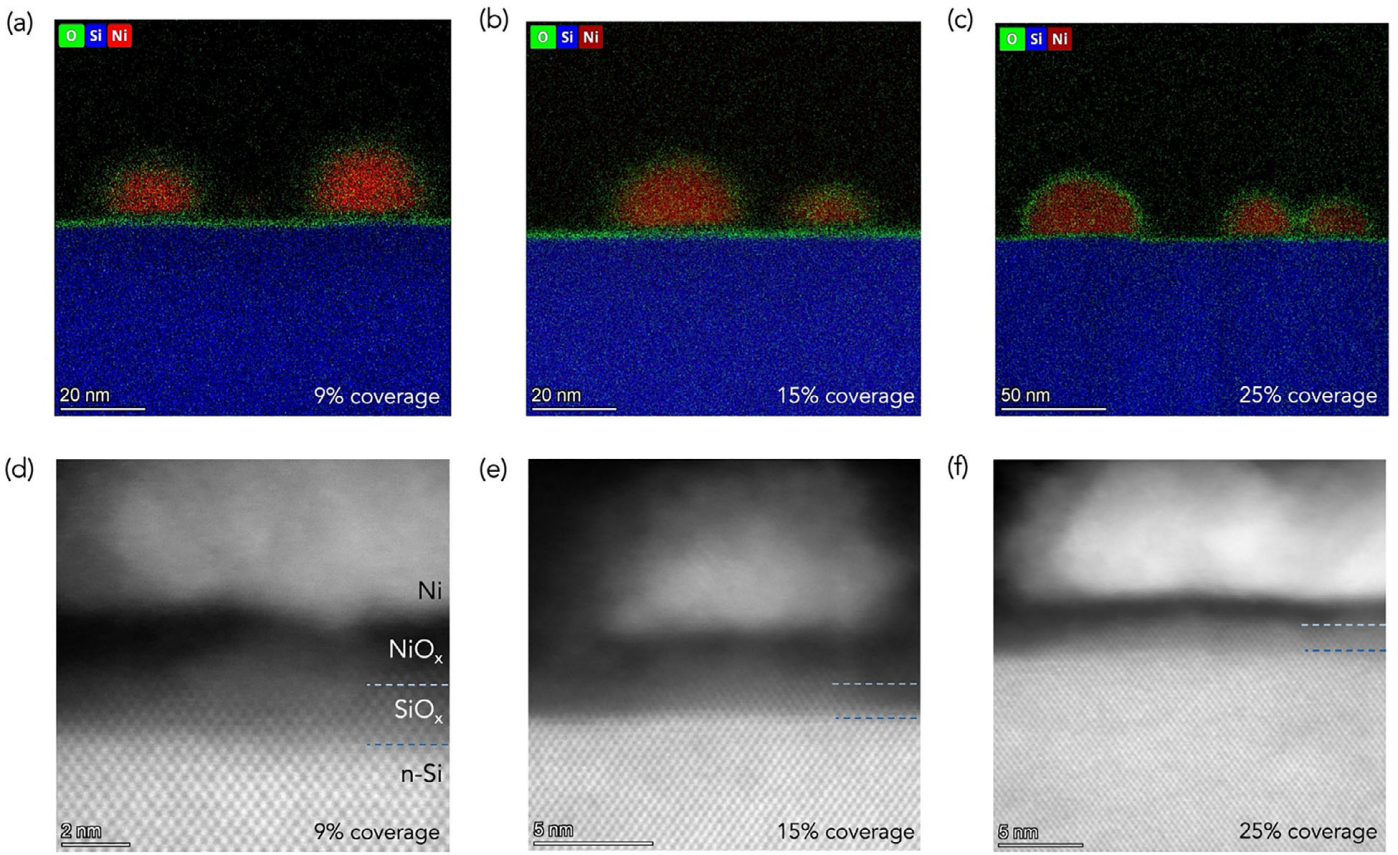
(a–c) Cross‐sectional STEM‐EDS images of (a) 9%, (b) 15%, and (c) 25% coverage samples post‐activation. (d–f) Atomic resolution cross‐sectional HAADF‐STEM images of the np‐Ni/SiO_x_/n‐Si interface for (d) 9%, (e) 15%, and (f) 25% coverage samples showing differences in the terminal interfacial SiO_x_ thickness. Dashed lines highlight the boundaries of the SiO_x_ layer.

To assess the operational stability of the photoanode and evaluate whether the in situ‐formed SiO_x_ interface evolves appreciably under extended operating conditions, we performed chronoamperometry (CA) on a representative np‐Ni/n‐Si photoelectrode with 18% surface coverage (Figure S7). We observed that the photovoltage remains stable over the first 6 h of operation, indicating that the interfacial SiO_x_ layer does not continue to grow appreciably during this period. After 6 h, performance begins to decline, with the 9 h CV indicating near‐complete deactivation. Possible contributors to this degradation include continued interfacial oxide evolution, Ni/NiO_x_ particle agglomeration, and catalyst delamination, though distinguishing between these mechanisms requires further investigation.

Based on these photochemical and geometric characterization studies, we hypothesized that several physical mechanisms could explain the differences in the measured photovoltage as a function of np‐Ni coverage on n‐Si.

- Increased photocurrent and light absorption due to smaller light reflection and parasitic absorption by metal np‐EC at lower coverage increases the concentration of photogenerated charge carriers in the SC. This increase in the number of charge carriers could lead to improvements in the photovoltage.
- A lower np‐EC surface coverage on SC reduces the direct SC/EC contact area, therefore limiting the transfer of majority charge carriers from SC to EC and, in doing so, lowering the parasitic e^−^/h^+^ recombination at the EC and increasing the photovoltage.
- Variations in the terminal interfacial SiO_x_ thickness between the np‐EC and SC, measured for different np‐Ni coverages, could also lead to the observed differences in photovoltage. It has been established that the performance of Ni/n‐Si systems is highly sensitive to the interfacial insulating oxide thickness [14, 27].

## 3 Analytical Modeling of the EC/SC Junction

To evaluate the hypotheses outlined above, we begin with the analytical illuminated diode expression that describes charge transfer (i.e., current) across metal‐insulator‐semiconductor (MIS) photoelectrodes as a function of applied voltage [28],

(1) $$J_{MIS}=J_{h}+J_{e}=J_{ph}\;-J_{s}\left[exp\left(-\frac{qV_{a}}{nkT}\right)-1\right]$$

Here, *J_ph_* is the photocurrent density (mA/cm^2^), which for an n‐type SC is equivalent to the total hole current density (*J_h_*) generated by solar absorption in the SC, *J_e_* is the total electron current density (mA/cm^2^) that reaches the metal EC, *q* is the elementary charge of an electron, *J_s_* is the reverse saturation current density (mA/cm^2^), *n* is the ideality factor, *k* is the Boltzmann constant, and *T* is the temperature (K). *V_a_* is the applied voltage defined as the potential of the majority charge carriers in the SC with respect to the solution potential. Assuming thermionic emission is the dominant recombination mechanism and that majority charge carriers have to tunnel through the insulating SiO_x_ layer in the np‐Ni/n‐Si system, the reverse saturation current, *J_s_*, can be described using

(2) $$J_{s}=f_{c}A^{*}\;T^{2}T_{t}exp\left(-\frac{q\phi_{b}}{kT}\right)$$

where *A* ^*^ is Richardson's constant, $\phi_{b}$ is the Schottky barrier height (including non‐idealities) [16], *f_c_* is the fractional interfacial contact area between np‐Ni and n‐Si, and *T_t_* is the tunneling probability.

The impact of an insulator layer between the EC and the SC is accounted for by the tunneling probability (*T_t_*) term. We note that for many oxides, such as HfO_2_ [14, 15, 16], Al_2_O_3_ [17, 27], and SiO_2_ [10, 11, 29] with thicknesses less than ∼ 3 nm, charge carriers tunnel through the oxide to reach the metal EC [30]. *T_t_* is a strong function of the thickness of the insulating layer. It can be captured by the following equation:

(3) $$T_{t}=\exp\left(-\propto d\sqrt{\phi_{e}}\right)$$

where ∝ is a constant that depends on the effective mass of electrons in the insulator, *d* is the insulator thickness, and $\phi_{e}$ is the offset between the SC and insulator conduction bands. The illustrated band diagram in Figure 3a summarizes the physical parameters used in the model.

**FIGURE 3:**
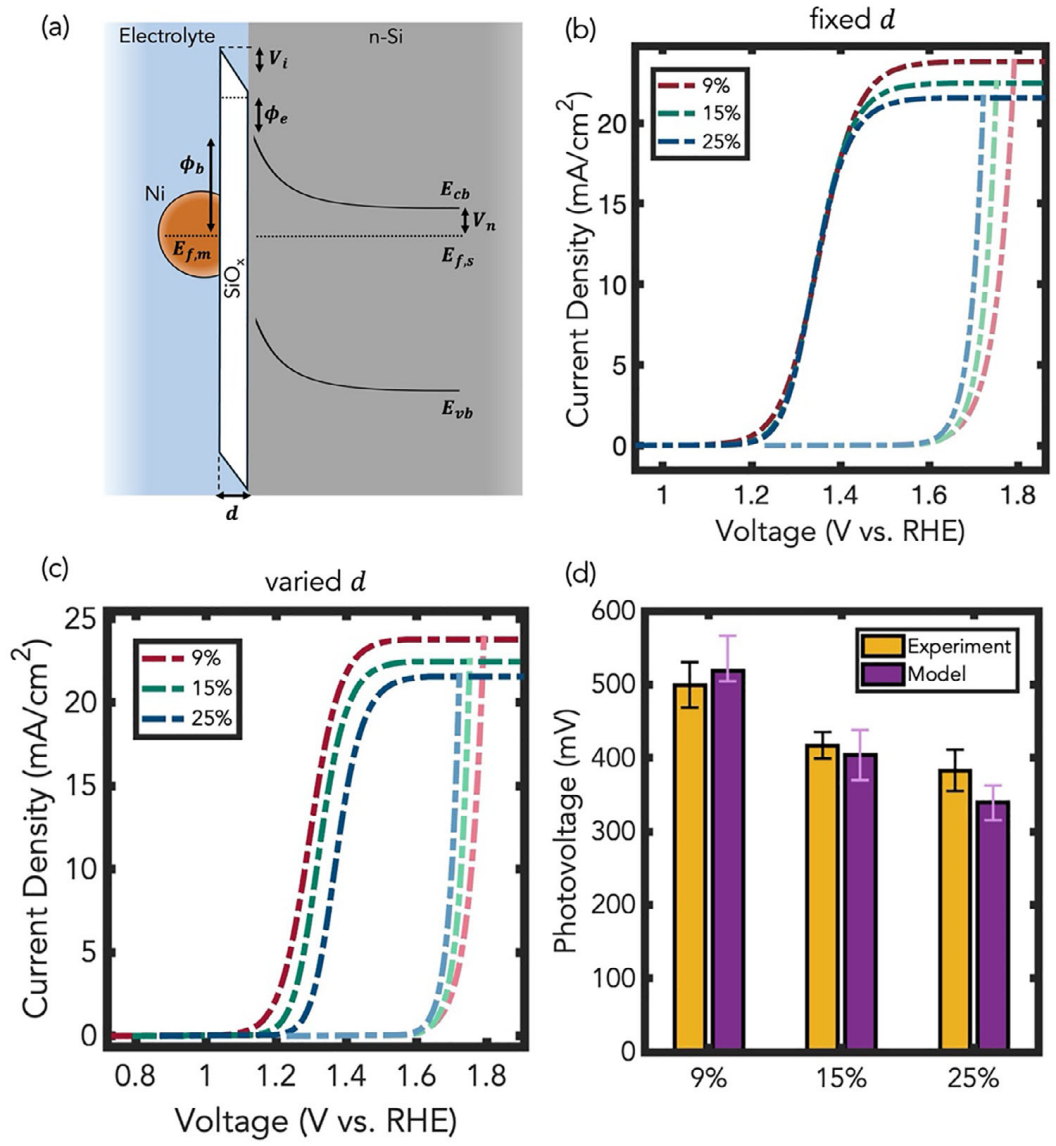
(a) Band diagram depicting the variables controlling charge transfer at the np‐MIS interface. *E_cb_*, *E_vb_*, *E*_*f*,*s*_, *E*_*f*,*m*_, *V_n_*, *V_i_*, $\phi_{e}$, $\phi_{b}$, and *d* are the conduction band edge, valence band edge, Fermi level of SC, Fermi level of metal, distance of Fermi level to the conduction band edge, voltage drop through the interface, tunneling barrier for electrons, barrier height and thickness of the insulator layer, respectively. Not drawn to scale. (b) Modeled light (solid dashed) and dark (faint dashed) *J–V* curves for all systems using experimental *f_c_*, *J_ph_*, and a constant SiO_x_ thickness of 2 nm. (c) Modeled light (solid dashed) and dark (faint dashed) *J–V* curves for all systems taking into account differences in the spontaneously evolved SiO_x_ thickness. (e) Comparison of measured and modeled photovoltages for all samples taking into account differences in the SiO_x_ layer thickness. Black and pink error bars show experimental error and model bounds, respectively.

We used the analytical framework presented above to model the photovoltage of each np‐Ni/n‐Si sample with a different np‐EC coverage (*f_c_*). First, *J_ph_* and *f_c_* values from the experimental data shown in Figure 1 for each coverage were input into Equations (1) and (2). As a baseline case, the interfacial oxide thickness (*d*) was set to a constant value of 2 nm. The intrinsic ideal Ni/n‐Si barrier height ($\phi_{b,ideal}$) was also assumed to be constant (0.67 V), consistent with previous measurements on Ni/NiO_x_/n‐Si junctions [11]. We generated complete current density–voltage (*J–V)* curves of photoelectrodes by coupling Equations ((1), (2), (3)) to the Butler–Volmer equation that describes the kinetics of OER (see Table S2 for details). Butler–Volmer kinetic parameters were extracted from the dark electrocatalytic activity of np‐Ni/p^+^‐Si samples shown in Figure 1a (accounting for varying EC coverage).

Figure 3b shows the modeled dark electrochemical *J–V* behavior together with the corresponding illuminated *J–V* curves derived from the analytical model. The dark fits capture the electrocatalytic response of the Ni EC and reflect the impact of catalyst coverage on the performance. As coverage decreases, the reduced catalytic surface area lowers the overall electrocatalytic activity, as indicated by the faint dashed curves in Figure 3b. On the other hand, the data in Figure 3b also show that modeled illuminated *J–V* curves for the three systems remain nearly identical (Figure 3b). This is inconsistent with the experimental data shown in Figure 1a, which show substantial differences between samples with varied coverage. Thus, the model result suggests that changes in catalyst coverage (*f_c_*) and light absorption (*J_ph_*) alone (hypotheses (i) and (ii)) cannot account for the experimentally observed trends in photoelectrochemical activity. This is because even though the model captures a slight increase in photovoltage due to increased *J_ph_* and reduced *f_c_*, this effect is counterbalanced by the simultaneous decrease in catalytic surface area, producing the data in Figure 3b.

Next, we tested hypothesis (iii), which suggests that variations in the interfacial SiO_x_ thickness across samples with different coverages could potentially explain the differences in photovoltages across samples. To assess this, we used the same analytical modeling framework described above. We note that in this case, the differences in *J_ph_* and *f_c_* were also taken into account. In addition, we allow the SiO_x_ thickness (*d*) to change in accordance with the experimentally measured values shown in Figure 2. The data in Figure 3c,d compare experimentally measured and analytically modeled *J–V* curves and photovoltages accounting for the differences in the interfacial SiO_x_ thickness. The data in Figure 3d show that the analytical model now accurately predicts the experimental photovoltage of all systems. Here, the upper and lower model bounds represent the modeled photovoltages, one standard deviation above and below the average SiO_x_ thickness measured. Figure S5 shows the experimental case studies overlaid with the fit for each coverage. Overall, the data show that there is excellent agreement between the modeled *J–V* curves and the experimental data. This result indicates that the analytical model reproduces the observed photovoltage differences between samples with varying catalyst coverage only when variations in the interfacial SiO_x_ thickness (*d*) are considered.

## 4 Origin of Coverage‐Dependent SiO_x_ Growth

Our physical and chemical characterization of photoanodes with varied nanoparticle EC coverage clearly established that np‐Ni/n‐Si systems with varying np‐Ni coverages evolve interfacial SiO_x_ with differing terminal thicknesses. Analytical modeling showed that the variations in SiO_x_ thickness are critical for accurately capturing the differences in photovoltage between these systems, indicating that this phenomenon plays a more significant role than variations in interface area and light absorption. The question that remains is why these samples evolve SiO_x_ to different thicknesses, given that they were fabricated and tested under identical conditions.

We hypothesized that there could be a difference in the total flux of photoexcited holes to the SC surface for photoelectrodes with different np‐Ni coverages. For low coverage samples, the number of interfacial EC/SC sites is low. This also implies that there will be a larger flux of holes to the SC surface for lower EC coverage samples, that is, the holes will end up at the SC surface as opposed to the EC, which could in theory lead to higher oxidation rates of Si to SiO_x_ [31, 32, 33, 34, 35].

To test this hypothesis, we performed COMSOL modeling to simulate the charge carrier concentration profiles for the EC/SC interfaces with varying coverages. Figure 4a shows the schematic of the 2D model geometry. The three cases we modeled included low (i.e., one particle), medium (i.e., two particles), and high (i.e., three particles) np‐EC coverages, corresponding to interfacial EC/SC coverages of 4% (low), 8% (medium), and 18% (high), respectively. In building the geometry, we maintained a constant SC slab size such that in each simulation the total number of charge carriers available in the bulk was identical. Experimentally determined parameters such as the photo‐limiting current, nanoparticle size, and so forth, were input into the model (see Table S3 for the list of model parameters).

**FIGURE 4:**
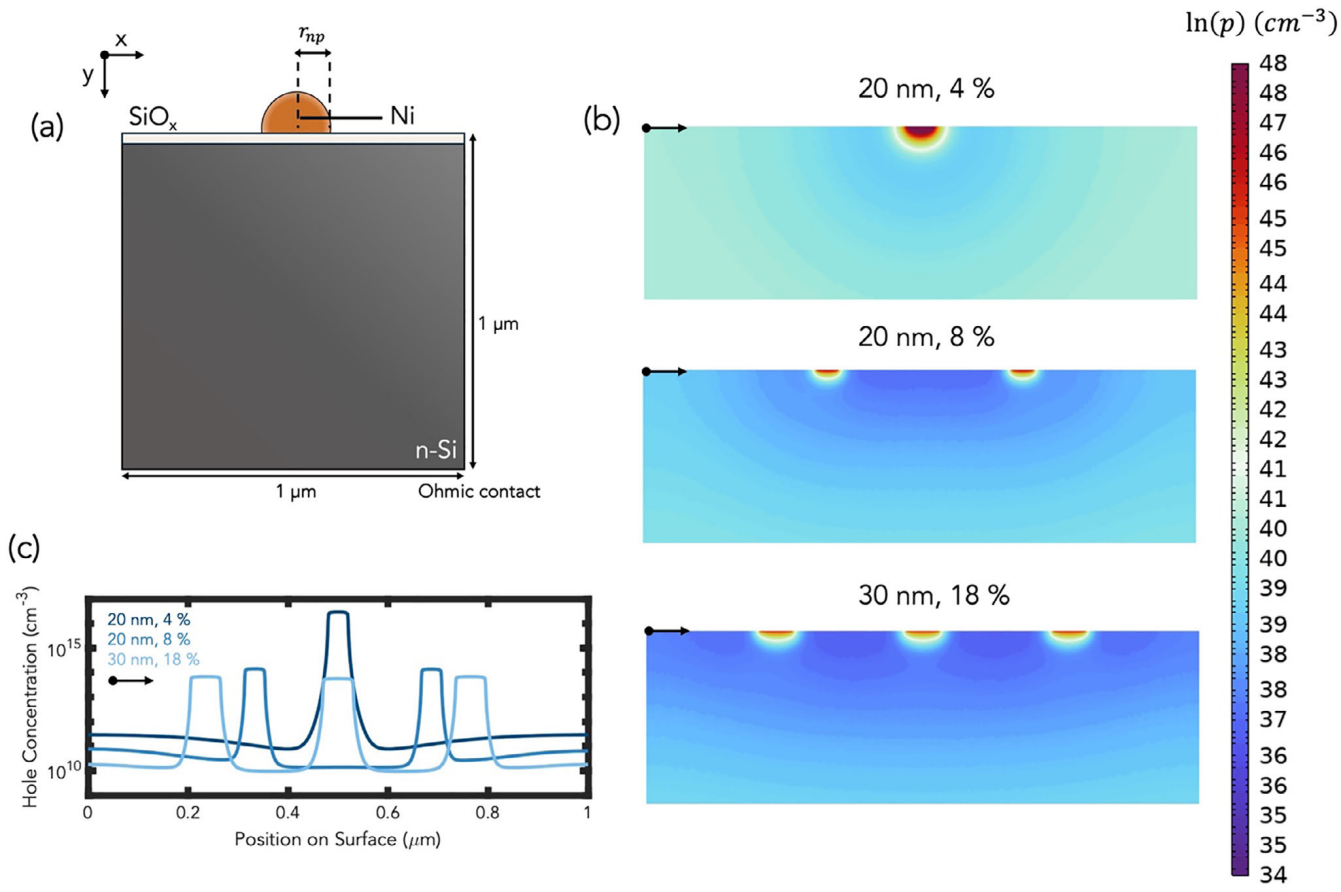
(a) COMSOL model geometry and dimensions of the np‐Ni/SiO_x_/n‐Si system. Governing equations were solved within the SC module. (b) Contour plot of the natural hole concentration (*p*) near the np‐Ni/n‐Si interface for systems with different interfacial coverage. Data shown at short‐circuit (*V*_a_ = 0 V) and the same SiO_x_ thickness (1.5 nm). (c) Surface plot of hole concentration for systems with different interfacial coverage. The ball and arrow show the position on the surface where the hole concentrations were calculated. See Figures S8 and S9 for additional simulation results.

The data in Figure 4b,c show the distribution and magnitude of hole concentrations near the SC surface for each photoelectrode under 1‐sun illumination intensity (at current densities matching the photo‐limiting current for OER in experiments). These data demonstrate that for lower np‐EC coverages (4%), the surface concentration of holes at the EC/SC interface and at the SC/electrolyte interface is higher than the hole concentrations at the same interfaces for medium (8%) and high (18%) coverages. This result is also supported by the electric field gradients present in each case study system, shown in Figure S10.

Overall, these results support our hypothesis that during the initial stages of potential cycling, excess holes at the np‐EC/SC and SC/electrolyte interface lead to a higher level of SiO_x_ growth for low coverage systems. We expect that this oxide growth leads to different terminal thicknesses and plateaus rather quickly as the photovoltage stabilizes between 10–30 potential cycles (see Figure S6). This behavior can be conceptually rationalized by considering the oxidation potential of the Si surface, which stabilizes at a different terminal SiO_x_ thickness depending on the np‐EC coverage due to differences in the surface concentration of photogenerated holes during operation [36].

While our results demonstrate that reducing np‐Ni/NiO_x_ coverage from 25% to 9% yields continued improvements in photovoltage, a coverage that maximizes overall PEC efficiency will depend on the balance between competing effects. At sufficiently low coverages, diminishing catalytic activity and potential over‐oxidation of exposed Si surface sites may offset the benefits of reduced parasitic light absorption and interfacial SiO_x_ formation. Exploring this regime systematically will require advanced fabrication techniques capable of producing well‐dispersed, size‐controlled nanoparticles at very low coverages, and represents a promising direction for future work.

## 5 Conclusions

The objective of this work was to evaluate the effect of varying nanoparticle coverage on photoelectrode performance. We establish that nanoparticle EC coverage not only dictates electrocatalytic activity and recombination rates but also governs the chemical evolution of the np‐EC/SC interface in np‐Ni/n‐Si model photoanodes for OER. Contrary to conventional expectations, reduced Ni coverage on n‐Si yields high photovoltages in large part because lower loadings drive the formation of thicker interfacial SiO_x_ under operating conditions. We attribute this effect to enhanced local hole accumulation at sparse interfaces, which accelerates surface SiO_x_ growth. These results highlight that in these systems, photovoltage improvements often arise not only from physical factors such as reduced optical losses or interface area alone, but also from dynamic chemical changes to the EC/SC interface. These findings point to a design space where optimal performance requires balancing catalyst loading, oxide evolution, and long‐term stability. More broadly, they underscore the need to account for reaction‐induced interface effects when engineering nanostructured photoelectrodes for efficient and durable solar‐to‐fuel conversion.

## Author Contributions

**Suljo Linic**: conceptualization, funding acquisition, Writing – review and editing, supervision, project administration, resources, data curation, Writing – original draft. **Ahmet Sert**: investigation, writing – original draft, methodology, validation, formal analysis, data curation, writing – review and editing. **Aarti Mathur**: investigation, writing – original draft, methodology, validation, writing – review and editing, formal analysis.

## Conflicts of Interest

The authors declare no conflicts of interest.

## Supporting information




**Supporting File**: adma74478‐sup‐0001‐SuppMat.docx.

## Data Availability

The data that support the findings of this study are available from the corresponding author upon reasonable request.
