# Supplementary material for: Electrocatalyst/Semiconductor Interfaces in Photoelectrocatalysts: Mechanistic Analysis of the Impact of Nanoparticle Electrocatalyst Coverage on Performance
Source: Adv Mater. 2026 Aug 4;38(56):e74478. doi: 10.1002/adma.74478 (PMC13648895; doi:10.1002/adma.74478)
Supplement: Supplementary file 1 [file ADMA-38-e74478-s001.docx]

**Supporting Information**

Electrocatalyst/Semiconductor Interfaces in Photoelectrocatalysts: Mechanistic Analysis of the Impact of Nanoparticle Electrocatalyst Coverage on Performance

Aarti Mathur^1,2^, Ahmet Sert^1,2^, and Suljo Linic^1,2*^

^1^Department of Chemical Engineering, University of Michigan, Ann Arbor, Michigan 48109, United States

^2^Catalysis Science and Technology Institute, University of Michigan, Ann Arbor, Michigan 48109, United States

^*^Lead contact: linic@umich.edu

**Experimental Section**

*Sample Fabrication*

Phosphorous doped (n-type, resistivity 0.1–1 ohm cm, (100)-oriented, 525 um thick) and boron doped (p^+^-type, resistivity 0.001–0.005 ohm cm, (100)-oriented, 525 µm thick) Si wafers were purchased from Addison Engineering. The Si wafers were hand-diced to 12 × 12 mm square pieces for all samples.

Fabrication procedure for np-Ni/Si was adapted from our prior work.^1^ Diced Si pieces were cleaned using a piranha solution (3/1 by volume concentrated aqueous H_2_SO_4_/30% aqueous H_2_O_2_) for 15 min and etched in buffered hydrofluoric acid (BHF) for 2 min. Ni was electrodeposited on wafer pieces using a solution of 0.01 M nickel chloride (NiCl_2_) and 0.1 M boric acid (H_3_BO_3_). NiCl_2_ and H_3_BO_3_ precursors were purchased from Millipore Sigma with 99.95% and 99.5% purity, respectively. The resulting solution was sonicated for 10 min and stirred for 5 min prior to electrodeposition. An Ag/AgCl reference electrode (Pine, FODR-0021) and Pt wire were placed in the electrodeposition solution. The position of the reference electrode was kept constant from sample to sample. Prior to loading, the back of the Si wafer piece was scratched using a diamond tip scribe, excess dust was removed, and gallium-indium eutectic paint (99.99%) was applied to the scratched area to ensure an ohmic contact. The prepared wafer piece was then housed in a custom 3D printed electrode with an O-ring and aperture of 0.459 cm^2^ exposed to the electrolyte. The back contact was placed against a Cu plate.

Once the electrode was submerged in the electrodeposition solution, EIS was used on each sample to evaluate the overall resistance, which includes the solution resistance and the resistivity from the wafers. The applied voltage was adjusted to its final value versus Ag/AgCl with a 5 s delay using a Gamry potentiostat. The applied voltage for electrodeposition was then selected by accounting for the ohmic losses such that the chronoamperometry plot is consistent from sample to sample and between n-type and p^+^-type samples. The chronoamperometry plots for the n-Si and p^+^-Si very closely overlapped which results in comparable nanoparticle sizes and distributions. The sample was then rinsed with DI water and immediately tested electrochemically. The average fabricated np-Ni/Si particle size and coverage for each regime were 34.2 ± 6.3 nm average particle radius with 21.1 ± 1.8% catalyst coverage (high), 28.9 ± 9.5 nm average particle radius with 14.1 ± 1.0% catalyst coverage (medium), and 22.1 ± 6.4 nm average particle radius with 7.3 ± 2.1% catalyst coverage (low). Nanoparticle size distributions were determined from scanning electron microscopy (SEM) image analysis, assuming circular particle geometry. Particle radii were binned into histograms to visualize the distribution of sizes for each sample.

*Electrochemical Testing*

For all electrochemical experiments, samples were scratched, painted, and housed in a 3D printed electrode using the same method as Ni electrodeposited samples described above. The illuminated area and the area exposed to the electrolyte were defined by an O-ring with an area of 0.459 cm^2^, and the current was normalized to this planar geometric area. All electrochemical experiments were performed using a three-electrode setup in a square quartz beaker.

For OER experiments, a 1 M KOH electrolyte prepared using 45 wt,% in H_2_O KOH (Millipore Sigma) and magnetically stirred. Samples were illuminated using a 300 W UV 16S-Series Solar Simulator (Solar Light Company) equipped with an AM 1.5 G filter. Light intensity was calibrated to 100 mW cm^-2^ (i.e., 1-sun) using a thermopile detector. n-type samples were illuminated during water oxidation measurements while p^+^-type samples were tested in the dark. A graphite rod counter electrode and Hg/HgO (Pine, RREF0038) reference electrode were used to complete the three-electrode setup. Cyclic voltammetry was conducted at scan rates between 50–200 mV s^–1^. For n-Si samples, voltages were typically cycled from −0.2 V up to about 0.9 V while for p^+^-Si, voltages were typically cycled from 0 V up to about 0.7 V (vs. Hg/HgO). Applied voltages were converted to RHE by calibrating the Hg/HgO reference electrode and accounting for the pH of the KOH solution:

$$\begin{aligned} V_{vs. RHE}= V_{vs. Hg/HgO}+V_{ref}+0.0591\cdot pH \#\left( S1 \right) \end{aligned}$$

For a typical set of OER experiments, the voltage was swept for a minimum of 30 cycles. Then a few additional voltage sweeps as well as EIS measurements were performed to evaluate the solution resistance. All *J*–*V* cyclic voltammogram plots were fully IR-corrected using the solution resistance. We note that while Ni/NiO_x_ nanoparticles with different coverages display differences in redox activity, including a shift in redox peak potential (Figure 1a), this reflects coverage-dependent differences in overpotential and Ni oxidation state rather than the redox peak position influencing interfacial SiO_x_ formation. This behavior is therefore not expected to affect the modeled interfacial properties.These tested samples were then rinsed with water and stored for SEM and STEM analysis.

The number of electrochemically active Ni sites was estimated by integrating the anodic peak of the forward CV sweep, corresponding to the Ni(OH)_2_ to NiOOH oxidation transition, using the relationship Q = nFN, where Q is the integrated charge, n is the number of electrons transferred per Ni atom (n = 1), F is Faraday's constant, and N is the number of active Ni atoms.  This approach is consistent with prior literature treating NiOOH as the catalytically active phase during OER.^2,3^ Across the coverage conditions studied herein, the estimated number of active Ni sites falls in the range of approximately 10^14^ to 10^16^ atoms per electrode, increasing systematically with NiO_x_ surface coverage.

*Sample Characterization*

Scanning electron microscopy (SEM) were performed using the TSF Nova 200 Nanolab with an accelerating voltage of 5 kV. Nanoparticle size, size distribution, and coverage were analyzed using the commercial IMAGEJ software. Scanning transmission electron microscopy (STEM) was performed with the TFS Talos F200X G2 to gather energy dispersive X-ray spectroscopy (EDS). TFL Spectra 300 probe-corrected microscope was used to gather high resolution interfacial images. STEM samples were prepared using focused ion-beam milling (FIB) on the TFS Nova 200 Nanolab.

Samples were tested under OER conditions for 30+ voltage sweeps and subsequent EIS prior to STEM preparation. Oxide thicknesses were measured using high-resolution STEM imaging where the Ni/NiO_x_/SiO_2_/Si interfaces were marked by changes in contrast and atomic lattice structure. The average thicknesses were determined by analyzing several nanoparticle samples with IMAGEJ software. To minimize confirmation bias, individual thickness measurements were recorded blindly across multiple locations on independent images before values were compiled and averaged. Layer boundaries were identified using contrast gradients and atomic lattice structure rather than visual inspection alone; the effective measurement resolution was constrained by the pixel size of the acquired images which determined both the precision of reported values and the estimated uncertainty.

*Analytical Modeling and COMSOL Simulations*

Complete details regarding analytical modeling techniques and determination of interfacial coverages from top-down SEM images can be found in our recent works.^1,4^ The analytical model is used to fully model the *J-V* curve of a photoanode. First, the dark electrochemical OER data obtained from np-Ni/p^+^-Si samples (Figure 1a) were fit to the Tafel equation to obtain the EC exchange current density ($J_{0,cat}$) and the Tafel slope ($b$):

$$\begin{aligned} J_{cat}= J_{0,cat}\left[ \exp\left( \frac{V_{cat}}{b} \right) \right]\#\left( S2 \right) \end{aligned}$$

Where $J_{cat}$ is the catalytic current density and $V_{cat}$ is the catalytic overpotential relative to the redox potential of OER. This equation is sufficient to model the *J-V* behavior of the EC only if the Tafel slope is constant throughout the potential range and if the series resistance has been subtracted (i.e., IR-compensation).

Then, the illuminated diode equation is used to model the *J-V* behavior of the photovoltage generating junction separately using the following equation:

$$\begin{aligned} J_{MIS}= J_{ph}- J_{s}\left[ \exp\left( -\frac{qV_{a}}{nkT} \right)-1 \right]\#\left( S3 \right) \end{aligned}$$

Where the $J_{MIS}$ is the MIS diode current density, $J_{ph}$ is the experimental photolimiting current density, $n$ is the ideality factor and $V_{a}$ is the applied voltage of the MIS diode. $J_{s}$ is calculated using Equation (2) in the main text. We note that $J_{ph}$ is measured directly from the experimental illuminated *J-V* curve and thus inherently captures the integrated effect of light absorption by the Si wafer and EC nanoparticles under operating conditions.^5,6^

Under the present assumptions, the catalytic current and the photovoltage generating diode (or solar cell) component of the system will be in series with each other. Therefore, the total current density through the system ($J_{total}$) is equal to the current density of each component:

$$\begin{aligned} J_{total}= J_{MIS}= J_{cat}\#\left( S4 \right) \end{aligned}$$

This implies that the applied voltage through each component will be additive:

$$\begin{aligned} V_{total}= V_{cat}+ V_{a} \#\left( S5 \right) \end{aligned}$$

We note that the ideality factor ($n$) influences the barrier height ($\phi_{b}$), since $n$ is directly linked to non-idealities at the interface (traps, defects, etc.) that lower $\phi_{b}$. These two variables are linked by the following relationship:

$$\begin{aligned} \phi_{b}= \frac{\phi_{b,ideal}}{n}+\left( \frac{n-1}{n} \right)V_{n} \#\left( S6 \right) \end{aligned}$$

Where $\phi_{b,ideal}$ is the ideal barrier height of the junction in the absence of non-idealities (provided in Table S1 below and $V_{n}$ is the difference in energy between the Fermi level and conduction band in the bulk of the SC (illustrated in Figure 2a).

Details related to the theory employed in the COMSOL modeling can be found in our recent work.^7^ The governing equations within the Semiconductor Module were solved using the MUMPS general solver in COMSOL Multiphysics 6.1 with a relative tolerance of 0.0001. The modeling domain was discretized with a non-uniform mesh of 800 elements with refinement near the metal EC/SC interfaces.

Table S1. Compiled experimental results for np-Ni/Si systems

|  | **Nanoparticle Radius (nm)** | | | **Interface Coverage (%)** | | | **Onset Potential (V vs. RHE)** | | | **SiO_x_ Thickness (nm)** | | **Photovoltage (mV)** | |
| --- | --- | --- | --- | --- | --- | --- | --- | --- | --- | --- | --- | --- | --- |
|  | n-Si | p^+^-Si | n-Si | | p^+^-Si | n-Si | | p^+^-Si | n-Si | | p^+^-Si | n-Si | p^+^-Si |
| **High Coverage** | 28.93 $\pm$ 0.38 | 39.46 $\pm$ 4.07 | 22.18 $\pm$ 1.19 | | 20.11 $\pm$ 2.00 | 1.246 $\pm$ 0.02 | | 1.629 $\pm$ 0.01 | 1.721 $\pm$ 0.125 | | - | 383 $\pm$ 28 | - |
| **Medium Coverage** | 26.86 $\pm$ 8.51 | 31.06 $\pm$ 11.9 | 13.99 $\pm$ 1.24 | | 14.28 $\pm$ 0.96 | 1.215 $\pm$ 0.01 | | 1.632 $\pm$ 0.01 | 1.937 $\pm$ 0.182 | | - | 417 $\pm$ 18 | - |
| **Low Coverage** | 16.52 $\pm$ 1.7 | 27.75 $\pm$ 2.54 | 6.88 $\pm$ 2.12 | | 7.75 $\pm$ 2.53 | 1.164 $\pm$ 0.02 | | 1.663 $\pm$ 0.01 | 2.461 $\pm$ 0.157 | | - | 498 $\pm$ 31 | - |

Table S2. List of model parameters used in the analytical model

| **Parameter** | **Symbol** | **Value** | **Units** | **Ref.** |
| --- | --- | --- | --- | --- |
| Temperature | $T$ | 298 | K | - |
| Relative permittivity | $\varepsilon_{r}$ | 11.7 | - | (^8^) |
| Richardson’s constant | $A^{*}$ | 120 | A/cm^2^ | (^8^) |
| Tunneling probability constant | $\propto$ | 1 | eV^1/2^ $Å$^-1^ | assumed |
| Photo-limiting current density | $J_{ph}$ | varied | mA/cm^2^ | measured |
| Si potential offset | $V_{n}$ | 0.211 | eV | (^9^) |
| Donor doping density | $N_{D}$ | 7.5 x 10^15^ | cm^-3^ | (^9^) |
| Ideality factor | $n$ | 1.77 | - | (^9^) |
| Interfacial SiO_x_ thickness | $d$ | varied | nm | measured |
| Nanoparticle top-down coverage | $f_{c,SEM}$ | varied | - | measured |
| Nanoparticle contact coverage | $f_{c}$ | $f_{c,SEM}/2$ | - | (^9^) |
| Insulator tunneling barrier | $\phi_{e}$ | 0.171 | eV | (^9^) |
| Ideal barrier height | $\phi_{b,ideal}$ | 0.67 | eV | (^9^) |
| Tafel slope | $b$ | varied | mV/decade | measured |
| Exchange Current Density | $J_{0,cat}$ | varied | mA/cm^2^ | measured |

**Discussion regarding the ideality factor**

Though not discussed in the main manuscript, another possible explanation for the observed changes in photovoltage with varying np-EC coverage is the variation in the ideality factor of the junction. One could think that if samples with lower np-Ni coverage form a more “ideal” EC/SC interface, the resulting decrease in the overall ideality factor could in theory reduce the voltage drop across the EC/SC interface, thereby increasing the photovoltage.

It is well established that the ideality factor and therefore the voltage drop across the insulator ($V_{i}$) tends to increase with increasing insulator thickness due to a higher density of interface states ($D_{s}$).^10–12^ Based on this, one would expect systems with lower catalyst coverage to exhibit higher ideality factors because they form thicker interfacial oxides, while higher-coverage samples would exhibit lower values. If this effect dominated, lower-coverage systems would be expected to experience a larger voltage drop across the insulator and therefore generate a lower photovoltage. Experimentally, we observed the opposite trend where the highest photovoltages occur for the lowest-coverage samples (Figure 1). This suggests that any increase in voltage drop associated with thicker oxides is largely offset by favorable tuning of charge-carrier fluxes through suppression of majority-carrier tunneling. Consequently, variations in the ideality factor cannot explain the observed photovoltage trends as a function of np-EC coverage. Moreover, in non-optimized MIS systems the ideality factor is typically in the range of 1.4-1.7, corresponding to a maximum voltage loss across the insulator of approximately ~70 mV, which is insufficient to account for the experimentally observed differences.^7,11,13^

**Figure S1.** (a) Representative electrodeposition chronoamperometry (CA) curves for np-Ni/n-Si samples with varying levels of electrocatalyst coverage, from the shortest deposition time and lowest coverage (yellow) to the longest deposition time and highest coverage (blue). (b) Particle size distribution histograms for np-Ni/n-Si and np-Ni/p^+^-Si samples, binned from particle radii measured by SEM image analysis.

**Figure S2.** Top-down SEM images of (a-c) np-Ni/n-Si and (d-f) np-Ni/p^+^-Si systems fabricated with varying coverage. (a), (d) high, (b), (e) medium, and (c), (f) low coverage, respectively. The scale bar in each image corresponds to 3 μm.

**Figure S3.** Cross-sectional STEM images and EDX maps of np-Ni/n-Si after 30+ cycles of electrochemical testing for 9% (a-d), 15% (e-h), and 25% (i-l) np-Ni/Si samples. Notably, the samples do not show any redeposition of Ni on the bare SiO_2_ surface after operation.

**Figure S4.** High resolution STEM dark-field (HAADF) images with top-down SEM coverages of (a) 9%, (b) 15%, and (c) 25%.

**Figure S5.** *J-V* plots for experimental systems (colored) and modeled fits (black dashed lines). (a) All three experimental case study systems (25%, 15%, 9%) magnified near the OER onset potential. (b-d) Full *J-V* model and experimental fits for (b) 9%, (c) 15%, and (d) 25% SEM coverage cases.

**Figure S6.** Evolution of a representative np-Ni/n-Si sample in the first 30 cycles of PEC OER. We note that the hypothesized oxide growth could continue with excess potential cycling (not explored here), causing the SiO_x_ layer to shift from a thin insulator to a thick insulator regime. However, in the thick (>3-4 nm) insulator regime, the flow of holes to the Ni EC will be inhibited by the low tunneling probability (as well as a large insulator potential drop), resulting in an overall decrease in photovoltage.^12,14^

**Figure S7: Chronoamperometric Stability Measurements**

**Figure S7:** Stability characterization of a representative np-Ni/n-Si photoanode with SEM-determined np-Ni surface coverage of 18%, subjected to 30 activation cycles prior to testing. (a) Cyclic voltammograms recorded at 2, 6, and 9 hours of operation, showing stable photovoltage over the first 6 hours followed by near-complete deactivation at 9 hours. (b) Chronoamperometry (CA) conducted at 1.5 V vs. RHE under 1 Sun illumination, with periodic interruptions at 2, 6, and 9 hours to remove evolved gas bubbles and record CVs. (c) Top-down SEM image of the representative electrode confirming 18% np-Ni surface coverage used for stability testing.

**Figure S8.** Impact of Ni nanoparticle contact spacing. (a-b) Model schematics for two Ni nanoparticle contacts with (a) 10 nm and (b) 300 nm particle spacing. (c) Modeled open-circuit photovoltages with varying particle spacing. The data shows that the particle spacing has no impact on the photovoltage generated at constant coverage. SiO_x_ thickness was 2 nm, photolimiting current density was 20 mA/cm^2^ and the ideality factor was assumed to be unity (n = 1) for both studies.

**Figure S9.** Modeled open-circuit photovoltages in COMSOL at different SiO_x_ thicknesses with varying coverages. Photolimiting current density was set to 20 mA/cm^2^ in all cases to isolate the impact of coverage and oxide thickness. Ideality factor was assumed to be unity (n = 1).

**Figure S10.** Plot of electric field norm for systems with varied coverage under model conditions identical to Figure 4b.

Table S3. List of parameters used in the COMSOL model

| **Parameter** | **Symbol** | **Value** | **Units** | **Ref.** |
| --- | --- | --- | --- | --- |
| Temperature | $T$ | 298 | K | - |
| Depth into semiconductor | $W$ | 1 | $\mu$m | - |
| Length of surface | $L$ | 1 | $\mu$m | - |
| Relative permittivity | $\varepsilon_{r}$ | 11.7 | - | (^8^) |
| Band gap | $E_{g}$ | 1.12 | eV | (^8^) |
| Electron affinity | $\chi$ | 4.05 | eV | (^8^) |
| Effective density of states in the conduction band | $N_{C}$ | 2.8 x 10^19^ | cm^-3^ | (^8^) |
| Effective density of states in the valence band | $N_{V}$ | 1 x 10^19^ | cm^-3^ | (^8^) |
| Electron mobility | $\mu_{n}$ | 1450 | cm^2^/(V$\cdot$s) | (^8^) |
| Hole mobility | $\mu_{p}$ | 500 | cm^2^/(V$\cdot$s) | (^8^) |
| Donor doping density | $N_{D}$ | 7.5 x 10^15^ | cm^-3^ | (^9^) |
| Radiative Recombination Rate Coefficient | $B$ | 10^-15^ | cm^3^/s | (^15^) |
| Nanoparticle radius | $r_{np}$ | 20 - 30 | nm | measured |
| Nanoparticle contact coverage | $f_{c}$ | 4 – 18 % | - | measured |
| Ni ideal work function | $\phi_{Ni,ideal}$ | 4.72 | eV | (^9^) |
| Richardson’s constant for electrons | $A_{n}^{*}$ | 120 | A/(K$\cdot$cm)^2^ | (^8^) |
| Richardson’s constant for holes | $A_{p}^{*}$ | 90 | A/(K$\cdot$cm)^2^ | (^8^) |
| Ideality factor | $n$ | 1 – 1.77 | - | (^9^) |
| Interfacial SiO_x_ thickness | $d_{ox}$ | 1.7 – 2.3 | nm | measured |
| Tunneling probability constant | $\propto$ | 1 | eV^1/2^ $Å$^-1^ | assumed |
| Insulator electron tunneling band offset | $\phi_{e}$ | 0.17 | eV | (^9^) |
| Photo-limiting current density | $J_{ph}$ | 20 - 22 | mA/cm^2^ | measured |

**References**

(1)	Hemmerling, J. R.; Mathur, A.; Linic, S. Characterizing the Geometry and Quantifying the Impact of Nanoscopic Electrocatalyst/Semiconductor Interfaces under Solar Water Splitting Conditions. *Advanced Energy Materials* **2022**, *12* (11), 2103798. https://doi.org/10.1002/aenm.202103798.

(2)	Mirabella, F.; Müllner, M.; Touzalin, T.; Riva, M.; Jakub, Z.; Kraushofer, F.; Schmid, M.; Koper, M. T. M.; Parkinson, G. S.; Diebold, U. Ni-Modified Fe3O4(001) Surface as a Simple Model System for Understanding the Oxygen Evolution Reaction. *Electrochimica Acta* **2021**, *389*, 138638. https://doi.org/10.1016/j.electacta.2021.138638.

(3)	Trotochaud, L.; Young, S. L.; Ranney, J. K.; Boettcher, S. W. Nickel–Iron Oxyhydroxide Oxygen-Evolution Electrocatalysts: The Role of Intentional and Incidental Iron Incorporation. *J. Am. Chem. Soc.* **2014**, *136* (18), 6744–6753. https://doi.org/10.1021/ja502379c.

(4)	Mathur, A.; Sert, A.; Linic, S. Common Misconceptions in the Analysis of Critical Figures of Merit for Functioning Electrocatalyst/Semiconductor Photoelectrocatalysts under Solar Water-Splitting Conditions. *ACS Energy Lett.* **2024**, *9* (8), 4136–4146. https://doi.org/10.1021/acsenergylett.4c01365.

(5)	Hernley, P. A.; Chavez, S. A.; Quinn, J. P.; Linic, S. Engineering the Optical and Catalytic Properties of Co-Catalyst/Semiconductor Photocatalysts. *ACS Photonics* **2017**, *4* (4), 979–985. https://doi.org/10.1021/acsphotonics.7b00047.

(6)	Trotochaud, L.; Mills, T. J.; Boettcher, S. W. An Optocatalytic Model for Semiconductor–Catalyst Water-Splitting Photoelectrodes Based on In Situ Optical Measurements on Operational Catalysts. *J. Phys. Chem. Lett.* **2013**, *4* (6), 931–935. https://doi.org/10.1021/jz4002604.

(7)	Sert, A.; Mathur, A.; Linic, S. Reactive Chemical Environments Control Charge Carrier Selectivity and Photovoltage at Nanoparticle Electrocatalyst/Semiconductor Junctions in Solar Water Splitting. *J. Am. Chem. Soc.* **2025**, jacs.5c07369. https://doi.org/10.1021/jacs.5c07369.

(8)	COMSOL Multiphysics® v. 6.1. COMSOL AB, Stockholm, Sweden.

(9)	Hemmerling, J. R.; Mathur, A.; Linic, S. Characterizing the Geometry and Quantifying the Impact of Nanoscopic Electrocatalyst/Semiconductor Interfaces under Solar Water Splitting Conditions. *Advanced Energy Materials* **2022**, *12* (11), 2103798. https://doi.org/10.1002/aenm.202103798.

(10)	Hemmerling, J.; Quinn, J.; Linic, S. Quantifying Losses and Assessing the Photovoltage Limits in Metal–Insulator–Semiconductor Water Splitting Systems. *Adv. Energy Mater.* **2020**, *10* (12), 1903354. https://doi.org/10.1002/aenm.201903354.

(11)	Hemmerling, J.; Mathur, A.; Linic, S. Consequences of Annealing on Metal–Insulator–Semiconductor Water Splitting Photoelectrocatalysts. *ACS Energy Lett.* **2024**, *9* (11), 5383–5390. https://doi.org/10.1021/acsenergylett.4c02337.

(12)	Hemmerling, J. R.; Mathur, A.; Linic, S. Design Principles for Efficient and Stable Water Splitting Photoelectrocatalysts. *Acc. Chem. Res.* **2021**, *54* (8), 1992–2002. https://doi.org/10.1021/acs.accounts.1c00072.

(13)	Laskowski, F. A. L.; Oener, S. Z.; Nellist, M. R.; Gordon, A. M.; Bain, D. C.; Fehrs, J. L.; Boettcher, S. W. Nanoscale Semiconductor/Catalyst Interfaces in Photoelectrochemistry. *Nat. Mater.* **2020**, *19* (1), 69–76. https://doi.org/10.1038/s41563-019-0488-z.

(14)	Scheuermann, A. G.; Lawrence, J. P.; Kemp, K. W.; Ito, T.; Walsh, A.; Chidsey, C. E. D.; Hurley, P. K.; McIntyre, P. C. Design Principles for Maximizing Photovoltage in Metal-Oxide-Protected Water-Splitting Photoanodes. *Nature Mater* **2016**, *15* (1), 99–105. https://doi.org/10.1038/nmat4451.

(15)	Wurfel, P.; Wurfel, U. *Physics of Solar Cells: From Basic Principles to Advanced Concepts*; Wiley - VCH, 2016.
